# Waist circumference glucose, a novel and effective predictor of type 2 diabetes: a prospective cohort study

**DOI:** 10.3389/fendo.2024.1427785

**Published:** 2024-07-29

**Authors:** Xiaofang Zhao, Bei Song, Tianci Yao, Haohui Fan, Ting Liu, Guangyu Gao, Kun Wang, Weilin Lu, Chengyun Liu

**Affiliations:** Department of Geriatrics, Union Hospital, Tongji Medical College, Huazhong University of Science and Technology, Wuhan, China

**Keywords:** type 2 diabetes mellitus, waist circumference, fasting plasma glucose, insulin resistance, Japan

## Abstract

**Introduction:**

Waist circumference (WC) and fasting plasma glucose (FPG) have been demonstrated as risk factors for type 2 diabetes mellitus (T2DM). Evidence is limited regarding the association of the combination of WC and FPG (WyG) with the risk of T2DM. The primary aim of the study was to investigate the relationship between WyG and T2DM.

**Research design and methods:**

The current study was a population-based cohort study using data from the NAGALA database. Participants were divided into tertiles based on WyG. Cox proportional hazard regression model was applied to identify the association of WyG with T2DM.

**Results:**

During a median follow-up of 6.19 years in the normoglycemia group and 5.58 years in the prediabetes group, respectively, 88 and 285 individuals in the two groups received a diagnosis of T2DM. After full adjustment, risk of T2DM increased in step-wise fashion with increasing tertiles of WyG. For a per-SD increase in WyG, the hazard ratios for T2DM were 3.05 (95% CI 2.64 - 3.51) in all populations, 1.94 (95% CI 1.46 - 2.58) in the normoglycemia group and 1.63 (95% CI 1.40 - 1.90) in the prediabetes group. The interaction between WyG and fatty liver on T2DM was statistically significant in the prediabetes group (*P* for interaction = 0.034).

**Conclusions:**

Elevated WyG was independently associated with incident T2DM in Japan. Baseline WyG help identify individuals at high risk of T2DM and implement effective preventive measures.

## Introduction

The prevalence of type 2 diabetes mellitus (T2DM) has increased considerably in recent decades. According to the International Diabetes Federation, diabetes will claim up to 700 million people worldwide by 2045, making it become one of the main threats to human health (1). Early identification and treatment of subjects at high risk for T2DM is critical, although its etiology and pathologic processes have not been fully elucidated (2). Given the large population of T2DM, it is more important to identify susceptible individuals early through simple and effective tools.

Insulin resistance (IR) is a central underpinning to the pathogenesis of T2DM (3–5). Hence, early diagnosis of IR is of paramount importance. Recent studies have shown that triglyceride glucose (TyG) is a widely used surrogate marker of IR and T2DM (6–8). In fact, TyG in T2DM and chronic complications is of interest for its simplicity, economy and high reproducibility. Notably, additional research has confirmed that triglyceride glucose-waist circumference (TyG-WC) offers a greater diagnostic value than TyG (2), which suggests that waist circumference (WC) is also of considerable importance. WC and fasting plasma glucose (FPG) are two well known risk factors for T2DM (9–11). However, the relationship of the combination of WC and FPG with T2DM is still unknown. On this basis, we propose a new index WyG (defined as ln [WC (cm) × FPG (mg/dL)/2]) based on the formula of TyG (defined as ln [1/2 TG (mg/dL) × FPG (mg/dL)]). The aims of the current study were to explore the relationship of WyG and T2DM.

## Materials and methods

### Data source and study cohort

This retrospective cohort study was based on data derived from the NAGALA (NAfld in the Gifu Area, Population-based Longitudinal Analysis) database, which has been described in detail elsewhere (12). All of the raw data are freely available from the DRYAD database (https://datadryad.org/). The full inclusion and exclusion criteria are detailed in the literature of the data source (12). Individual who participated in the medical examination program at Murakami Memorial Hospital from 2004 to 2015 were included in the study. Exclusion criteria for this cohort included T2DM at baseline or fasting plasma glucose ≥ 6.1 mmol/L, missing data, known liver disease, ethanol consumption (> 60 g/day for men and 40 g/day for women) and medication usage. The NAGALA database has received ethical approval from the Ethics Committee of Murakami Memorial Hospital. All participants in this project have signed informed consent to use their data for research (12).

### Exposure and covariates

The study exposure was WyG. WyG was calculated as ln [WC (cm) × FPG (mg/dL)/2].

Baseline demographic, clinical and laboratory characteristics were collected. The study variables were as follows: age, sex, body mass index (BMI), WC, alanine aminotransferase (ALT), aspartate aminotransferase (AST), gamma-glutamyl transferase (GGT), total cholesterol (TC), triglycerides (TG), high-density lipoprotein cholesterol (HDL), FPG, glycosylated hemoglobin (HbA1c), systolic blood pressure (SBP), diastolic blood pressure (DBP), fatty liver, and follow up duration. All participants completed a questionnaire on their use of tobacco and alcohol, and exercise habits.

### Outcome

The main outcome of the study was the new-onset T2DM, which was diagnosed according to one of the criteria (FPG ≥ 7 mmol/L, HbA1c ≥ 6.5%, or self-reported) (13).

### Statistical analyses

The total cohort was divided into two groups according to the following criteria: prediabetes is defined by FPG ≥ 100mg/dL or 5.7% ≤ HbA1c < 6.5%; normoglycemia is defined by FPG < 100mg/dL or HbA1c < 5.7% (14).

First of all, participant baseline characteristics by the WyG tertiles were compared using analysis of variance or the Kruskal-Wallis test for continuous variables and the chi-squared test for categorical variables. We then applied a univariate analysis model to estimate the relation between the baseline characteristics and T2DM. In addition, Cox proportional hazards models and restricted cubic spline (RCS) analysis were applied to explore the potential relationship between WyG and T2DM. Subsequently, we further analyzed the potential diagnostic value of WyG in T2DM by using receiver operating characteristic (ROC) curve analysis and informativeness analysis (15). Finally, the interaction between baseline WyG and the risk of T2DM were performed among subgroups stratified as gender, fatty liver, smoking status (never, past, current), alcohol consumption (non, light, moderate, heavy), and habit of exercise.

All calculations were performed using EmpowerStats (www.empowerstats.com, X&Y solutions, Inc., Boston MA) and R statistical software (http://www.R-project.org). *P* < 0.05 (two-tailed) was considered to be statistically significant.

## Results

### Characteristics of study subjects

**Figure 1:**
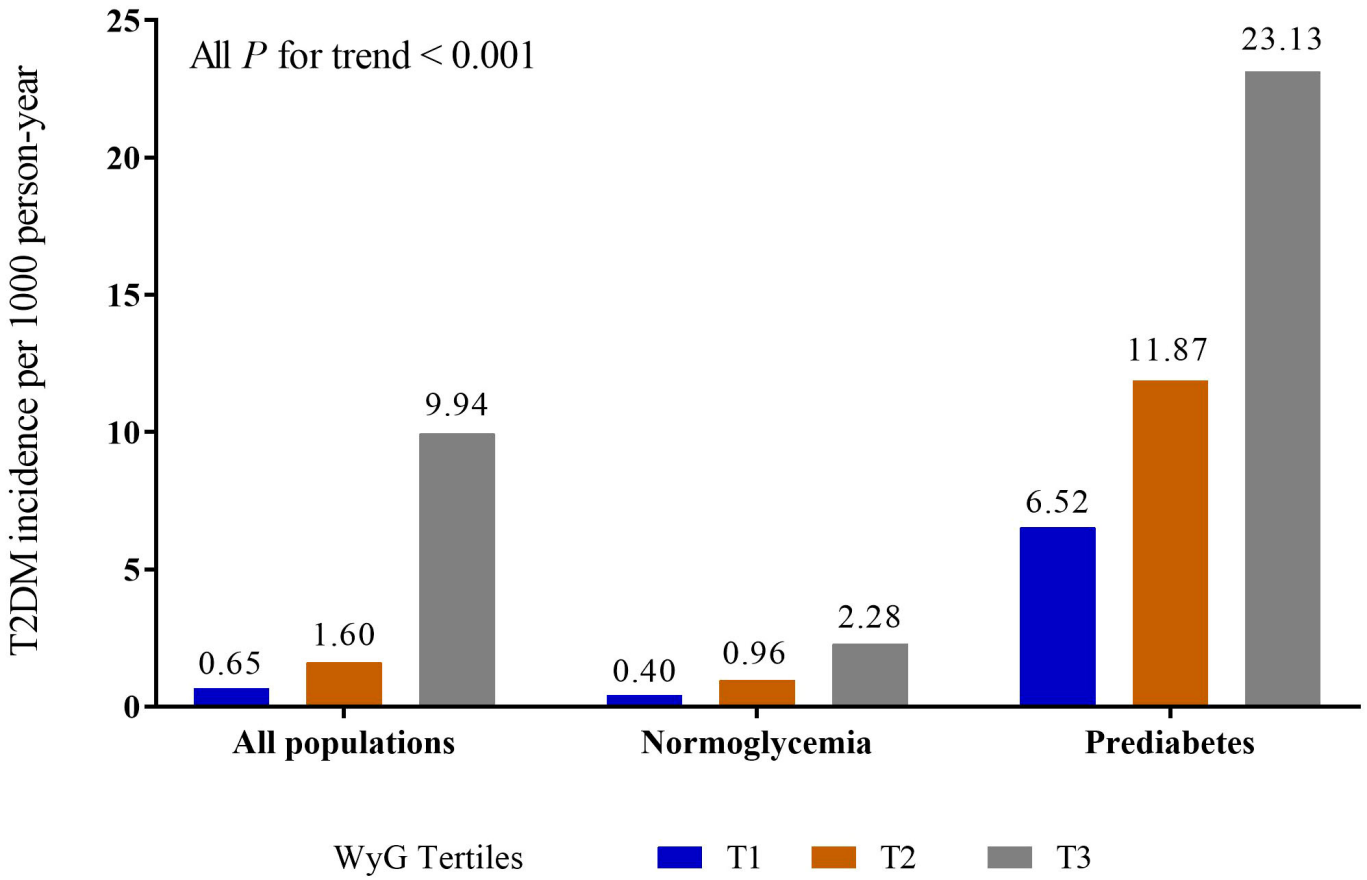
Incidence of T2DM in different populations. WyG, waist circumference glucose; T2DM, type 2 diabetes mellitus.

**Table 1** showed the baseline characteristics of the study in all populations according to WyG tertiles. The values of BMI, WC, ALT, AST, GGT, TC, TG, HbA1c, FPG, SBP, and DBP levels were highest and the HDL was lowest in the group with the highest tertile of WyG. The proportions of male, older people, subjects with fatty liver, smokers (past or current), drinkers (light or moderate or heavy) and without habit of exercise were highest in the group with the highest tertile of WyG. Additionally, the group with the WyG highest tertile had the highest incidence of T2DM (9.94, 2.28 and 23.13 cases per 1000 person-years among all populations, the normoglycemia group and the prediabetes group, respectively) (**Figure 1**).

**Table 1:** Baseline characteristics of participants according to WyG tertiles.

Variable	WyG			*P*-value
	Tertile 1	Tertile 2	Tertile 3	
	(7.13 - 8.09)	(8.09 - 8.25)	(8.25 - 8.81)	
Sample size	5155	5153	5156	
Age, yrs	41.61 ± 8.59	43.89 ± 8.78	45.63 ± 8.87	<0.001
BMI, kg/m^2^	19.60 ± 1.83	21.86 ± 1.97	24.89 ± 2.83	<0.001
WC, cm	67.60 ± 4.90	76.25 ± 4.52	85.57 ± 6.54	<0.001
ALT, IU/L	14.00 (11.00-17.00)	16.00 (13.00-21.00)	22.00 (17.00-31.00)	<0.001
AST, IU/L	16.00 (13.00-19.00)	17.00 (14.00-21.00)	19.00 (15.00-24.00)	<0.001
GGT, IU/L	12.00 (10.00-15.00)	15.00 (12.00-21.00)	21.00 (15.00-32.00)	<0.001
TC, mg/dl	190.18 ± 32.29	197.33 ± 32.36	207.10 ± 33.39	<0.001
HDL, mg/dl	64.36 ± 14.99	56.46 ± 14.74	48.80 ± 12.82	<0.001
TG, mg/dl	46.00 (34.00-64.00)	66.00 (46.00-94.00)	95.00 (65.00-139.00)	<0.001
HbA1c, %	5.09 ± 0.30	5.15 ± 0.31	5.27 ± 0.33	<0.001
FPG, mg/dl	86.67 ± 5.57	93.04 ± 5.21	99.19 ± 5.41	<0.001
SBP, mmHg	106.35 ± 12.57	114.41 ± 13.09	122.73 ± 14.47	<0.001
DBP, mmHg	66.03 ± 8.84	71.35 ± 9.36	77.37 ± 10.06	<0.001
Sex				<0.001
Women	4145 (80.41%)	2026 (39.32%)	863 (16.74%)	
Men	1010 (19.59%)	3127 (60.68%)	4293 (83.26%)	
Fatty liver				<0.001
No	5088 (98.70%)	4610 (89.46%)	3025 (58.67%)	
Yes	67 (1.30%)	543 (10.54%)	2131 (41.33%)	
Smoking status				<0.001
Never	4000 (77.59%)	2878 (55.85%)	2153 (41.76%)	
Past	468 (9.08%)	1038 (20.14%)	1446 (28.04%)	
Current	687 (13.33%)	1237 (24.01%)	1557 (30.20%)	
Alcohol consumption				<0.001
No	4528 (87.84%)	3860 (74.91%)	3417 (66.27%)	
Light	353 (6.85%)	667 (12.94%)	738 (14.31%)	
Moderate	233 (4.52%)	444 (8.62%)	683 (13.25%)	
Heavy	41 (0.80%)	182 (3.53%)	318 (6.17%)	
Habit of exercise				<0.001
No	4259 (82.62%)	4175 (81.02%)	4321 (83.81%)	
Yes	896 (17.38%)	978 (18.98%)	835 (16.19%)	

Continuous variables are presented as mean (SD) or median (25th, 75th percentile), and categorical variables are presented as number (percentage).

WyG, waist circumference glucose; BMI, body mass index; WC, waist circumference; ALT, alanine aminotransferase; AST, aspartate aminotransferase; GGT, gamma-glutamyl transferase; TC, total cholesterol; HDL, high-density lipoprotein cholesterol; TG, triglycerides; HbA1c, glycosylated hemoglobin; FPG, fasting plasma glucose; SBP, systolic blood pressure; DBP, diastolic blood pressure.

### Baseline variables and T2DM

**Figure 2:**
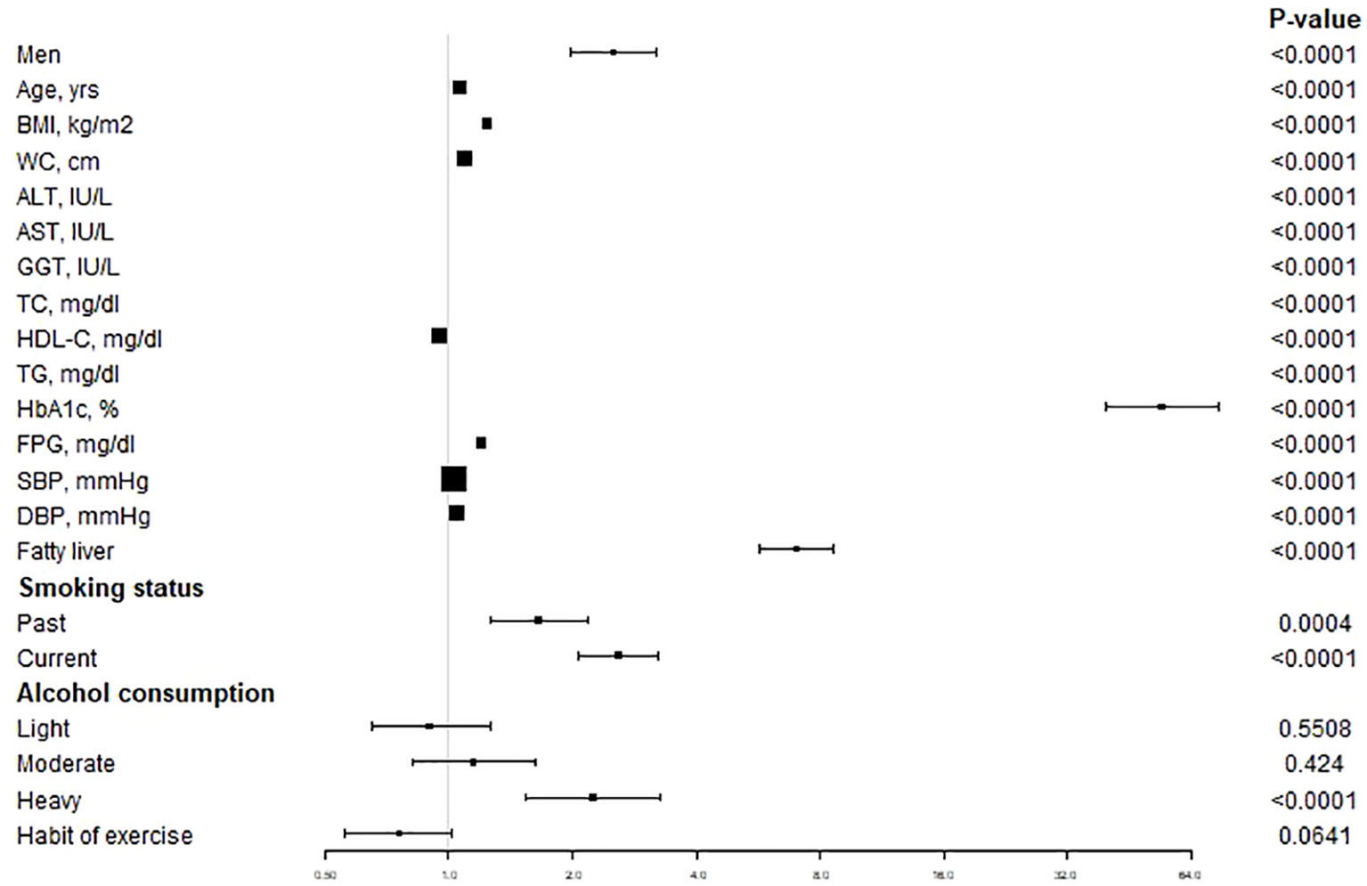
The unadjusted association between baseline variables and incident T2DM. Data are Hazard ratios and 95% CI. T2DM, type 2 diabetes mellitus; BMI, body mass index; WC, waist circumference; ALT, alanine aminotransferase; AST, aspartate aminotransferase; GGT, gamma-glutamyl transferase; TC, total cholesterol; HDL-C, high-density lipoprotein cholesterol; TG, triglycerides; HbA1c, glycosylated hemoglobin; FPG, fasting plasma glucose; SBP, systolic blood pressure; DBP, diastolic blood pressure.

**Figure 2** showed the relation between baseline characteristics in all populations and the risk of T2DM. The results demonstrated that male, age, BMI, WC, ALT, AST, GGT, TC, TG, HbA1c, FPG, SBP, DBP, fatty liver, smoking status (past or current) (*P* < 0.05) and alcohol consumption (heavy) were positively correlated with the risk of T2DM and that the HDL (*P* < 0.0001) was negatively correlated with the risk of T2DM.

### Independent relation between WyG and T2DM

**Table 2** showed the results of the multivariate Cox proportional hazards regression analysis. Compared with the referent first tertile of WyG, the hazard ratios (HRs) of incident T2DM for the second and third tertiles increased in the unadjusted, minimally adjusted (adjusted for age and sex) and fully adjusted (adjusted for age, sex, ALT, GGT, TC, fatty liver, smoking status, alcohol consumption and habit of exercise) models. In all populations, the HRs of incident T2DM were 2.23 (95%CI, 1.31 - 3.79) in the second tertile of WyG, and 8.79 (95%CI, 5.31 - 14.55) in the third tertile of WyG in the fully adjusted model. In the normoglycemia group, the HRs of incident T2DM were 2.16 (95%CI, 0.99 - 4.72) in the second tertile of WyG, and 3.08 (95%CI, 1.35 - 7.03) in the third tertile of WyG in the fully adjusted model. In the prediabetes group, the HRs of incident T2DM were 1.41 (95%CI, 0.95 - 2.10) in the second tertile of WyG, and 2.03 (95%CI, 1.36 - 3.05) in the third tertile of WyG in the fully adjusted model. Furthermore, we also found that WyG per-SD change was positively correlated with the risk of T2DM in all groups (HR: 3.05 in all populations; HR: 1.94 in the normoglycemia group; HR: 1.63 in the prediabetes group; all *P* < 0.0001).

**Table 2:** Associations of baseline WyG with incident T2DM.

	Crude model		Minimally model		Fully model	
	Hazard ratio (95%CI)	*P*-value	Hazard ratio (95%CI)	*P*-value	Hazard ratio (95%CI)	*P*-value
All populations						
WyG Per-SD increase	3.79 (3.38, 4.26)	<0.0001	4.05 (3.57, 4.58)	<0.0001	3.05 (2.64, 3.51)	<0.0001
WyG Tertiles						
T1	Reference		Reference		Reference	
T2	2.52 (1.51, 4.21)	0.0004	2.64 (1.56, 4.45)	0.0003	2.23 (1.31, 3.79)	0.0031
T3	15.64 (10.05, 24.35)	<0.0001	16.41 (10.17, 26.5)	<0.0001	8.79 (5.31, 14.55)	<0.0001
*P* for trend	<0.0001		<0.0001		<0.0001	
Normoglycemia						
WyG Per-SD increase	2.45 (1.96, 3.07)	<0.0001	2.57 (1.99, 3.31)	<0.0001	1.94 (1.46, 2.58)	<0.0001
WyG Tertiles						
T1	Reference		Reference		Reference	
T2	2.52 (1.20, 5.30)	0.0147	2.37 (1.10, 5.13)	0.028	2.16 (0.99, 4.72)	0.0537
T3	5.91 (3.01, 11.60)	<0.0001	5.29 (2.47, 11.34)	<0.0001	3.08 (1.35, 7.03)	0.0074
*P* for trend	<0.0001		<0.0001		0.008	
Prediabetes						
WyG Per-SD increase	1.98 (1.73, 2.26)	<0.0001	2.12 (1.85, 2.43)	<0.0001	1.63 (1.40, 1.90)	<0.0001
WyG Tertiles						
T1	Reference		Reference		Reference	
T2	1.78 (1.23, 2.57)	0.0023	1.96 (1.33, 2.88)	0.0007	1.41 (0.95, 2.10)	0.0874
T3	3.50 (2.50, 4.90)	<0.0001	3.95 (2.74, 5.71)	<0.0001	2.03 (1.36, 3.05)	0.0006
*P* for trend	<0.0001		<0.0001		0.0002	

Statistical analysis method used: cox regression analysis.

WyG, waist circumference glucose; T2DM, type 2 diabetes mellitus.

Crude model adjusted for: none.

Minimally model adjusted for: sex; age.

Fully model adjusted for: sex; age; alanine aminotransferase; gamma-glutamyl transferase; total cholesterol; fatty liver; alcohol consumption; smoking status; habit of exercise.

**Figure 3:**
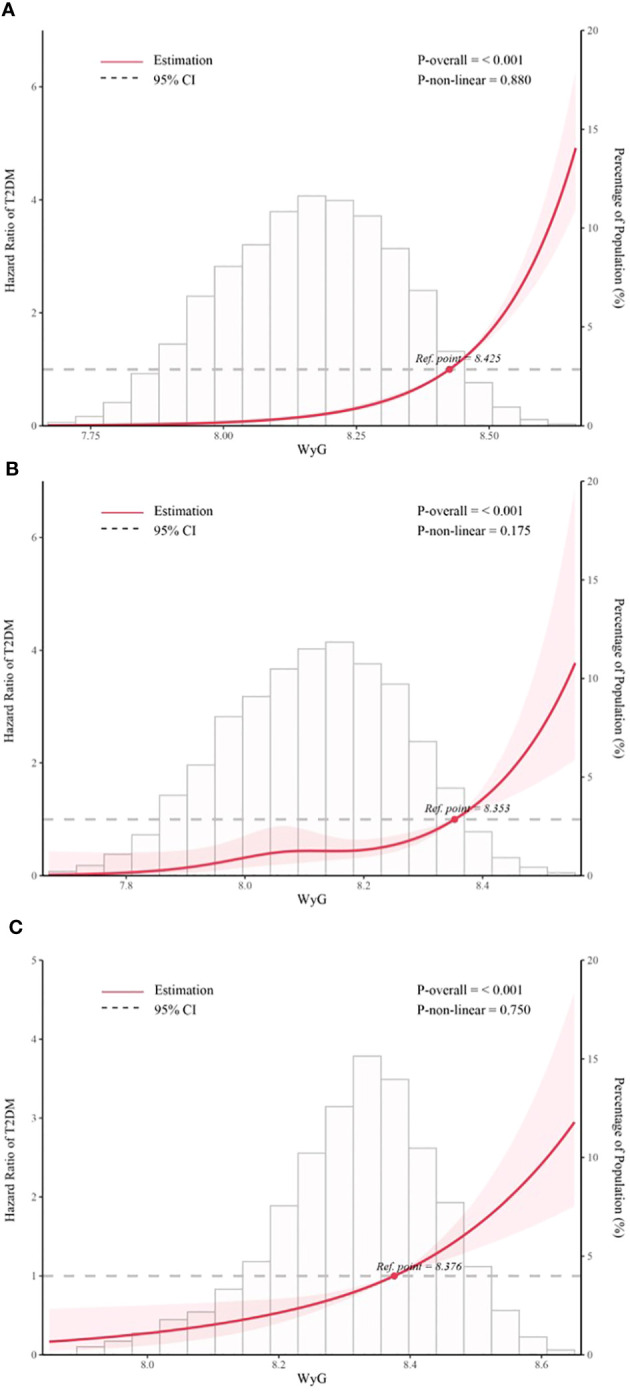
Five knots restricted cubic spline (RCS) plots of adjusted dose–response relationships for WyG and the risk of T2DM in different populations, with density plots indicating the distribution of WyG. **(A)** all populations, **(B)** normoglycemia, **(C)** prediabetes. WyG, waist circumference glucose; T2DM, type 2 diabetes mellitus. All models were adjusted for sex, age, alanine aminotransferase, gamma-glutamyl transferase, total cholesterol, fatty liver, alcohol consumption, smoking status, habit of exercise.

We then went on to explore the potential nonlinear relationship between WyG and T2DM by the RCS analysis. The results (**Figure 3**) revealed a linear relationship between WyG and new-onset T2DM in all populations, normoglycemia and prediabetes (all *P* for nonlinearity > 0.05).

### Predictive efficacy of WyG for new-onset T2DM

**Figure 4:**
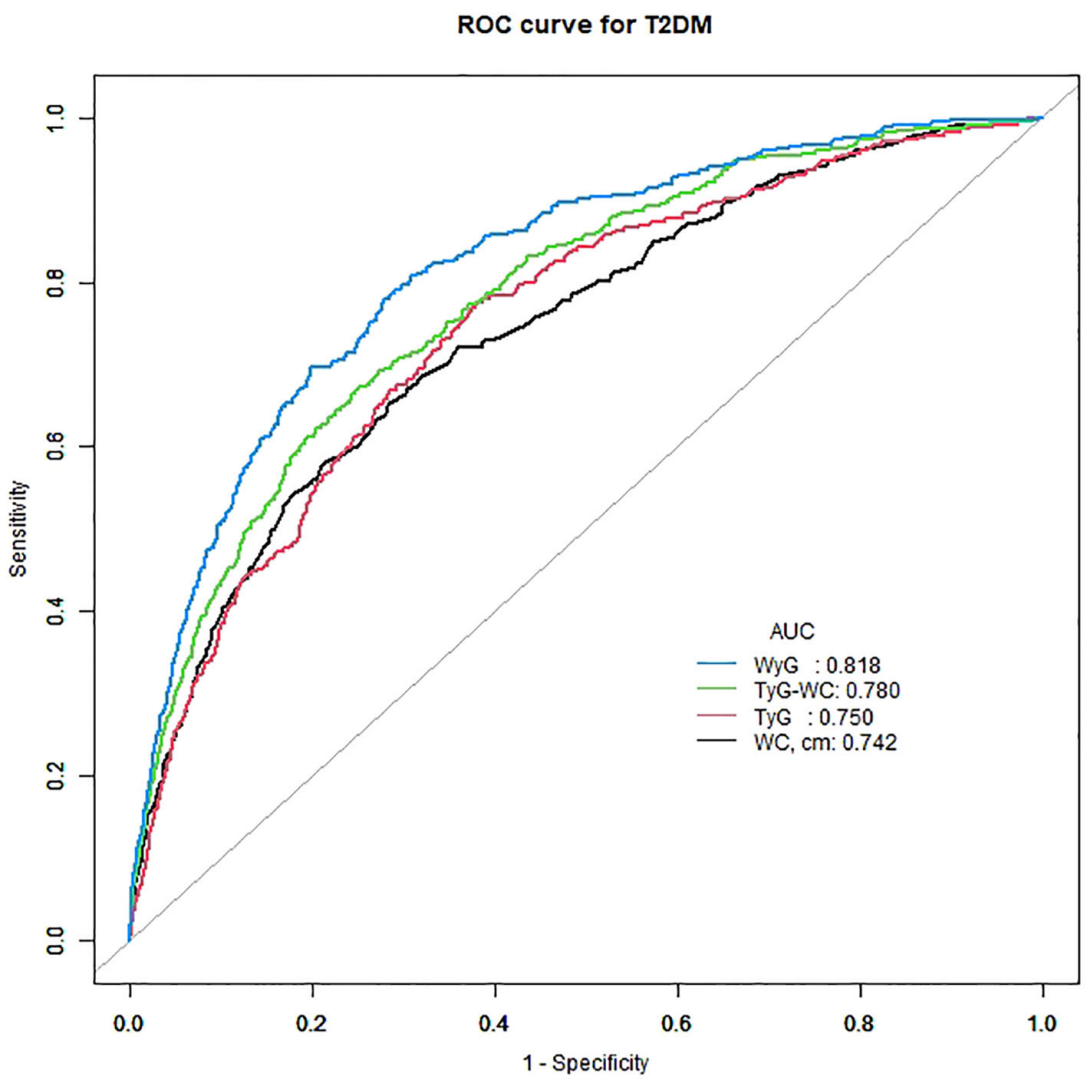
ROC curve analysis of the relationship between different indices and T2DM. T2DM, type 2 diabetes mellitus; WC, waist circumference; TyG, triglyceride glucose; TyG-WC, triglyceride glucose-waist circumference; WyG, waist circumference glucose.

The areas under the ROC curves (AUCs) were used to evaluate the effectiveness of WyG in predicting the risk of T2DM in all populations (**Figure 4**). The result showed that WyG (AUC 0.818) was the best marker for identifyingT2DM when compared with WC (0.742), TyG (AUC 0.750) and TyG-WC (AUC 0.780). Similar to this result, WyG was the most informative measure for the risk of T2DM (its relative informativeness compared with TyG and TyG-WC was 128%; **Table 3**).

**Table 3:** Relative informativeness of different indices for T2DM.

	Confounder adjusted χ² statistic	Informativeness*
TyG	512.4	100%
TyG-WC	512.4	100%
WyG	653.8	128%

*Informativeness of the given measure (as indicated by the confounder-adjusted χ2 statistic relating it to T2DM), as a percentage of the informativeness of TyG.

T2DM, type 2 diabetes mellitus; TyG, triglyceride glucose; TyG-WC, triglyceride glucose-waist circumference; WyG, waist circumference glucose.

Models adjusted for sex; age; alanine aminotransferase; gamma-glutamyl transferase; total cholesterol; fatty liver; alcohol consumption; smoking status; habit of exercise.

### Association between WyG and T2DM in subgroups

We stratified the analysis by sex, fatty liver, smoking status (never, past, current), alcohol consumption (non, light, moderate, heavy) and habit of exercise, which further supported that WyG was highly positively correlated with T2DM (**Table 4**). There was no interaction between above subgroups in all populations and the normoglycemia group (all *P*_interaction_ > 0.05). Interestingly, there was a significant interactive effect of WyG and fatty liver on T2DM in the prediabetes group (*P*_interaction_ = 0.034).

**Table 4:** Association of WyG with incident T2DM in subgroups.

	No. of events	Hazard ratio (95% CI)	*P*-value	*P* for interaction
All populations				
Sex				0.6733
Female	7034	3.07 (2.39, 3.95)	<0.0001	
Male	8430	3.05 (2.55, 3.64)	<0.0001	
Fatty liver				0.2352
No	12723	2.68 (2.15, 3.35)	<0.0001	
Yes	2741	3.22 (2.66, 3.89)	<0.0001	
Smoking status				0.6439
Never	9031	3.21 (2.58, 3.99)	<0.0001	
Past	2952	2.66 (1.83, 3.86)	<0.0001	
Current	3481	3.06 (2.43, 3.86)	<0.0001	
Alcohol consumption				0.6068
No	11805	3.08 (2.61, 3.63)	<0.0001	
Light	1758	2.66 (1.68, 4.23)	<0.0001	
Moderate	1360	2.64 (1.52, 4.57)	0.0005	
Heavy	541	-^a^		
Habit of exercise				0.151
No	12755	2.96 (2.54, 3.45)	<0.0001	
Yes	2709	4.02 (2.64, 6.12)	<0.0001	
Normoglycemia				
Sex				0.2975
Female	6031	2.33 (1.47, 3.68)	0.0003	
Male	5775	1.66 (1.14, 2.43)	0.0084	
Fatty liver				0.3658
No	10280	1.69 (1.17, 2.46)	0.0054	
Yes	1526	2.35 (1.48, 3.74)	0.0003	
Smoking status				0.277
Never	7281	2.48 (1.64, 3.73)	<0.0001	
Past	1985	1.50 (0.67, 3.36)	0.3196	
Current	2540	1.62 (1.00, 2.61)	0.0508	
Alcohol consumption				0.2265
No	9283	1.98 (1.45, 2.70)	<0.0001	
Light	1252	0.94 (0.28, 3.17)	0.9242	
Moderate	919	1.49 (0.44, 5.10)	0.5242	
Heavy	352	-^a^		
Habit of exercise				0.6727
No	9739	1.90 (1.40, 2.58)	<0.0001	
Yes	2067	2.47 (1.10, 5.57)	0.0293	
Prediabetes				
Sex				0.4674
Female	1003	1.59 (1.22, 2.08)	0.0006	
Male	2655	1.66 (1.37, 2.02)	<0.0001	
Fatty liver				0.034
No	2443	1.27 (0.99, 1.64)	0.0623	
Yes	1215	1.80 (1.49, 2.17)	<0.0001	
Smoking status				0.4493
Never	1750	1.63 (1.30, 2.06)	<0.0001	
Past	967	1.37 (0.93, 2.03)	0.1129	
Current	941	1.84 (1.43, 2.36)	<0.0001	
Alcohol consumption				0.27
No	2522	1.74 (1.46, 2.08)	<0.0001	
Light	506	1.25 (0.77, 2.03)	0.3609	
Moderate	441	1.03 (0.57, 1.86)	0.9136	
Heavy	189	-^a^		
Habit of exercise				0.306
No	3016	1.60 (1.36, 1.89)	<0.0001	
Yes	642	2.02 (1.28, 3.17)	0.0025	

Data were adjusted for sex; age; alanine aminotransferase; gamma-glutamyl transferase; total cholesterol; fatty liver; alcohol consumption; smoking status; habit of exercise.

WyG, waist circumference glucose; T2DM, type 2 diabetes mellitus.

a The model failed because of the small sample size.

## Discussion

### Principal findings

In this study, we found that exposures to elevated WyG was associated with an increased risk of incident T2DM. We also noted a dramatic difference across fatty liver subgroup in the prediabetes group. In the fatty liver group, an increase in WyG led to a significant increase in the risk of T2DM (HR: 1.80; 95% CI: 1.49 - 2.17; *P* < 0.0001), but no such result was found in the non-fatty liver group (*P* = 0.0623). Our results may suggest that WyG was a good predictor of T2DM when compared to WC, TyG and TyG-WC.

### Comparison with other studies

Several indicators have been reported to improve T2DM screening efficiency. Oral glucose tolerance test is still too expensive and time-consuming to be used routinely. A previous study showed that a combination of HbA1c, FPG and WC was effective in screening for individuals at risk for future T2DM (16). The role for the combined measurement of FPG and HbA1c for the prediction of T2DM has been confirmed in multiple studies (17, 18). One study on gestational diabetes mellitus revealed that the combination of HbA1c and WC had relatively high sensitivity to detect T2DM (19). Our study differed from previous studies in several important aspects. On the one hand, our results established for the first time that there was a positive correlation between the combination of WC and FPG and T2DM. On the other hand, in our analyses, we simultaneously attend to the data from all populations, the normoglycemia cohort and the prediabetes cohort.

Previous studies have shown that IR was considered the main pathophysiological component of diabetes (3–5). Increasingly, research has considered the role of TyG in IR and diabetes. TyG not only was used to assess IR and diabetes, but also can better predict diabetes than the homeostatic model assessment of IR (6–8). Accordingly, TyG has become a widely accepted marker of diabetes. Besides, a recent study has confirmed that TyG-WC, as a TyG-related index, was a better risk marker for diabetes than TyG (2). This implied WC played a key role in predicting diabetes risk. WC was a representative of central obesity. In the National Cholesterol Education Program-Adult Treatment Panel-III, central obesity was recognized as an independent risk factor for diabetes (20). WC by itself or with other indicators are critical factors of predicting diabetes risk (21, 22). On this basis, we invented a new indicator – WyG, which combined WC and FPG together. Moreover, WyG is much easier to perform than TyG-WC. Importantly, WyG had higher predictive performance for the risk of T2DM than WC, TyG and TyG-WC. Therefore, it is necessary to further understand and explore WyG.

### Meaning of the study

The strong link between obesity and diabetes has long been established (5, 23, 24). Obesity was also tightly linked to FPG (25). In addition, reactive oxygen species produced by adipose tissue may cause a variety of metabolic disorders such as obesity-related IR and T2DM (26). Besides obesity, the independent risk factors for T2DM also included FPG (27). Our results suggested that WyG, as a combination of WC and FPG, can predict diabetes. This was predominantly due to the well-documented role of obesity and FPG in the development of IR and diabetes. Besides, elevated glucose has a toxic effect upon beta cells by increasing reactive oxygen species (28). Taken together, these results suggested that the role of glucotoxicity and lipotoxicity in the pathogenesis of diabetes cannot be neglected.

Furthermore, compared with TG, WC was inexpensive, simple and non-invasive. Conveniently, an accurate ruler can measure WC in real time at home. WyG further simplified the calculation of TyG-WC. Owing to its convenience and simplicity, WyG was applicable not only to large-scale health assessment, but also to individuals’ evaluation of themselves.

Notably, there was a significant interactive effect of WyG and fatty liver on T2DM in the prediabetes group. Our results, if confirmed, suggest that individuals with high WyG benefit from the treatment of fatty liver to prevent T2DM. Obesity and IR are well known to be key pathogenic factors for both fatty liver and T2DM (29). The treatment of obesity and IR in fatty liver might decrease the risk of developing T2DM by reducing FPG and WC.

### Strength and limitations of this study

This study has some notable strengths. The large size and long follow-up period of the cohort study enhanced the reliability of the results. More importantly, compared with previous studies, we adjusted for more confounding factors to further increase the credibility of the results. Besides, given that the data come from Japan, the results were more instructive to the Japanese. More meaningfully, WyG was first proposed as a new predictor of T2DM. Simultaneously, WyG can be added to the reference biomarkers during follow-up of T2DM.

This analysis also has limitations. Firstly, the study remained limited to mainly single-center analyses in Japan, rendering the results not applicable to other regions and ethnicities. Evidence based on different regions and ethnic groups was needed to support the generality of the findings. Then, the prevalence of diabetes may be inaccurate on account of the lack of oral glucose tolerance tests. Finally, unmeasured confounding factors may not be fully addressed given that the study data were from an existing database. We were unable to obtain plasma Sirtuin 1 and insulin levels, which were important to identify individuals with T2DM (30–32).

## Conclusions

Our study first found a positive association of baseline WyG with incident T2DM in Japan. It highlighted the importance of the treatment of fatty liver to prevent T2DM, especially in people with prediabetes. Monitoring WyG may have utility in identifying individuals at increased risk of T2DM. Given this, it is critical that the underlying mechanisms of these associations receive further study.

## Data availability statement

The datasets presented in this study can be found in online repositories. The names of the repository/repositories and accession number(s) can be found below: The datasets generated during and/or analyzed during the current study are available in the dryad database, https://datadryad.org/stash/dataset/doi:10.5061%2Fdryad.8q0p192.

## Ethics statement

The studies involving humans were approved by the ethics committee of Murakami Memorial Hospital. The studies were conducted in accordance with the local legislation and institutional requirements. Written informed consent for participation was not required from the participants or the participants’ legal guardians/next of kin in accordance with the national legislation and institutional requirements.

## Author contributions

XZ: Conceptualization, Formal Analysis, Investigation, Validation, Writing – original draft, Writing – review & editing. BS: Conceptualization, Investigation, Writing – original draft. TY: Data curation, Writing – review & editing. HF: Data curation, Writing – review & editing. TL: Data curation, Methodology, Writing – original draft. GG: Methodology, Writing – original draft. KW: Data curation, Writing – original draft. WL: Conceptualization, Project administration, Supervision, Writing – review & editing. CL: Conceptualization, Funding acquisition, Project administration, Resources, Supervision, Writing – review & editing.
